# Optimization of oxalic acid pre-treatment and enzymatic saccharification in *Typha latifolia* for production of reducing sugar

**DOI:** 10.1186/s43141-020-00042-w

**Published:** 2020-07-09

**Authors:** Sunil Kodishetty Ramaiah, Girisha Shringala Thimappa, Lokesh Kyathasandra Nataraj, Proteek Dasgupta

**Affiliations:** 1 Bioenergy Lab, Department of Biotechnology, Bangalore University, Bengaluru, Karnataka 560056 India; 2 Department of Biotechnology, M.S. Ramaiah Institute of Technology, Bengaluru, Karnataka 560054 India; 3 Department of Zoology, Bangalore University, Bengaluru, Karnataka 560056 India

**Keywords:** Optimization, Oxalic acid, Pre-treatment, Reducing sugar, Saccharification, *Typha latifolia*

## Abstract

**Background:**

Plants with high biomass can be manipulated for their reducing sugar content which ultimately upon fermentation produces ethanol. This concept was used to enhance the production of reducing sugar from cattail (*Typha latifolia*) by oxalic acid (OAA) pre-treatment followed by enzymatic saccharification.

**Result:**

The optimum condition of total reducing sugar released from OAA pre-treatment was found to be 22.32 mg/ml (OAA—1.2%; substrate concentration (SC)—6%; reaction time (RT)—20 min) using one variable at a time (OVAT). Enzymatic saccharification yielded 45.21 mg/ml of reducing sugar (substrate concentration (SC)—2.4%; enzymatic dosage—50 IU/g; pH 7.0; temp—50 °C) using response surface methodology (RSM).

**Conclusion:**

We conclude that *Typha* can be used as a potential substrate for large-scale biofuel production, employing economical bioprocessing strategies.

## Background

Renewable biofuel from agricultural biomass is a replacement option for fossil fuel depletion and can overcome problems in energy security and economy. Yet, there are problems in performing large-scale biofuel production like technical knowledge and economic investment [3]. Aquatic weeds have high biomass content and grow uncontrollably in wetlands; hence, they can be used as a potential source for bioethanol production as opposed to agricultural crops that require monetary investment. Cattail (*Typha latifolia*) has high cellulose content constituted by polysaccharides and low cell wall integrity which could ease hydrolysis. Cellulose matrix is covered with lignin and other polysaccharides which can be converted into sugars by pre-treatment and enzymatic saccharification [22, 31].

Lignocellulosic biomass is a complex molecule surrounded by lignin and hemicellulose which resist enzymatic hydrolysis [24]. The conversion of cellulose to sugar requires the biomass to undergo oxalic acid pre-treatment which decreases the structural components by disrupting the lignin and crystalline cellulose [19], thereby enhancing enzymatic saccharification of the substrate. Therefore, the present study efforts to optimize pre-treatment and enzymatic saccharification in *Typha*, for maximal yield of reducing sugars by using one variable at a time (OVAT) technique and response surface methodology (RSM). The sugars produced can further be fermented to yield bioethanol, a source of biofuel.

## Method

### Sample collection

Matured plants (182–213 cm tall) were collected during spring-summer transition (March–April 2017) from Yelahanka Lake (28–30 °C), Karnataka. The shoots and leaves were washed, chopped (2–4 cm), dried (70 °C; 48 h), ground to 2 mm (particle size) and stored in a glass container according to the protocol of Waghmare et al. [28]. The formal identification of the plant material was conducted by the University of Agricultural Sciences, Bangalore. A voucher specimen was deposited in the publicly available herbarium (deposition number—UASB4606).

### Cellulase enzyme source

Crude cellulase enzyme was obtained from culture broth of *Bacillus cereus* (MH 590292) isolated from soil sample, was grown in Czapek-Dox medium for 24 h under optimum conditions (45 °C, pH 7.0) and agitated at 120 rpm. The supernatant was collected for purification and further experimentation.

### Cellulase enzyme purification

The culture broth was purified by the fractional ammonium sulphate precipitation method [5]. Supernatant collected previously was mixed with ammonium sulphate (80% saturation range), gently stirred (20 min; 4 °C) and incubated for 4–8 h. After precipitation, the mixture was centrifuged at 10,000×*g* at 4 °C for 15 min. The supernatant was discarded, and the pellet was mixed with 0.02 M Tris HCL buffer solution (pH 8.0). The crude enzyme in the buffer solution was taken into a dialysis bag (maintained at 4 °C) with 20 mM Tris buffer solution and changed twice. The enzyme mixture was then lyophilized, freeze-dried to powder and was used as a crude cellulase enzyme source for further experimentation.

### Pre-treatment of biomass

Pre-treatment was performed by different concentrations of oxalic acid (0.4–2.0% w/w), substrate (2.0–8.0% w/v) and reaction time (5–30 min), for yield of sugars. Since pre-treatment was performed in autoclave, temperature and pressure were kept constant at 121 °C under 15 psi [11]. Oxalic acid concentration, substrate concentration and reaction time were optimized by OVAT.

### Design of experiments by RSM

Central composite design (CCD) was used to optimize the enzymatic hydrolysis of biomass [10]. Four independent variables, pH, temperature, substrate concentration and enzyme dosage were selected for this purpose (Table 1). Measured response and independent variables are represented in second-order polynomial equation (Eq. 1).

1 $$ Y={\beta}_0+{\beta}_1{x}_1+{\beta}_2{x}_2+{\beta}_3{x}_3+{\beta}_4{x}_4+{\beta}_{11}{x}_1^2+{\beta}_{22}{x}_2^2+{\beta}_{33}{x}_3^2+{\beta}_{44}{x}_4^2+{\beta}_{55}{x}_5^2+{\beta}_{12}{x}_1{x}_2+{\beta}_{13}{x}_1{x}_3+{\beta}_{14}{x}_1{x}_4+{\beta}_{23}{x}_2{x}_3+{\beta}_{24}{x}_2{x}_4+{\beta}_{34}{x}_3{x}_4 $$

**Table 1:** The central composite design with four independent variables and the experimental results (C*—control)

Run no.	Substrate concentration (%w/v) *X*_1_	Enzyme loading (IU/g) *X*_2_	pH *X*_3_	Temp (°C) *X*_4_	Reducing sugars (mg/g) observed value	Reducing sugars (mg/g) predicted value
1	1.2	30	04	35	12.85	10.07
2	1.2	30	04	65	19.27	20.24
3	1.2	30	10	35	15.06	15.09
4	1.2	30	10	65	24.54	21.68
5	1.2	70	04	35	16.92	17.09
6	1.2	70	04	65	20.83	19.86
7	1.2	70	10	35	15.62	13.93
8	1.2	70	10	65	13.74	13.12
9	3.6	30	04	35	28.27	28.10
10	3.6	30	04	65	30.02	28.72
11	3.6	30	10	35	33.72	31.70
12	3.6	30	10	65	29.69	28.74
13	3.6	70	04	35	39.92	39.78
14	3.6	70	04	65	33.81	33.00
15	3.6	70	10	35	36.95	35.20
16	3.6	70	10	65	25.06	24.85
17	0.5	50	07	50	10.45	13.26
18	4.8	50	07	50	45.21	47.23
19	2.4	10	07	50	22.18	24.83
20	2.4	90	07	50	26.82	27.95
21	2.4	50	01	50	14.28	14.91
22	2.4	50	13	50	8.62	11.77
23	2.4	50	07	20	21.02	23.31
24	2.4	50	07	80	21.64	23.13
25C*	2.4	50	07	50	15.10	12.76
26C*	2.4	50	07	50	11.47	12.76

*Y* Reducing sugar production in milligram/gram

*β*_0_ Regression coefficient of the model

*β*_1_, *β*_2_, *β*_3_ Linear effect of independent variables

*β*_11_, *β*_22_, *β*_33_ Square effect of independent variables

*β*_12_, *β*_13_, *β*_14_, *β*_23_, *β*_24_, *β*_34_ Interaction effect of selected independent variables

### Statistical analysis

The CCD model data was analysed with Statistica 13.4 (TIBCO Software Inc. CA, USA). The statistical inference drawn was based on analysis of variance (ANOVA) and *F* test. The quality of second-order polynomial regression equation was assessed by regression of coefficient (*R*^2^). Further, the model predicted optimal levels of independent variables which were tested in triplicates, and the observed value was finally compared with the predicted value.

### Optimization of enzymatic saccharification

Experiments were carried out in Erlenmeyer flasks (50 ml) containing sodium citrate buffer (50 mM; 20 ml) and sodium azide (2%) to prevent microbial contamination. All the parameters like substrate concentration, pH, temperature and enzyme dosage range were setup using RSM [25]. After 24 h of enzymatic saccharification, the samples were taken from the fermentable broth and centrifuged at 10,000×*g* for 5 min, the supernatant withdrawn for analysis of reducing sugars. The CCD model was used to optimize the effects of temperature (°C), pH, cellulase load (IU/g) and substrate concentration (%w/v) on enzymatic saccharification of cattail to obtain high sugar yield. In total, 26 experiments were designed (Table 1) for yield of reducing sugar and obtained by the following regression equation:

2 $$ Y=22.60-5.22{x}_1+3.40{x}_1^2-0.38{x}_2+0.008{x}_2^2+2.44{x}_3+0.16{x}_3^2-0.39{x}_4+0.001{x}_4^2+0.04{x}_1{x}_2-0.09{x}_1{x}_3-0.13{x}_1{x}_4-0.03{x}_2{x}_4-0.006{x}_2{x}_4-0.019{x}_3{x}_4 $$

*Y* Response of reducing sugar yield

*X*_1_ Independent variable of substrate concentration

*X*_2_ Enzyme load

*X*_3_ pH

*X*_4_ Temperature

### Estimation of reducing sugar (DNS method)

The amount of total reducing sugar was determined by 3,5-dinitrosalicylic acid method [18]. Sample (1 ml) was mixed with 3,5-dinitrosalicylic acid (DNS) (2 ml) reagent in the test tube and immersed in boiling water bath for 5 min. The sample was cooled at room temperature and absorbance measured at 540 nm. The amount of reducing sugar liberated was calibrated by using standard glucose curve and expressed as milligram/milliliter.

### Cellulase enzyme activity

Crude cellulase enzyme (0.5 ml) was mixed with 1% carboxymethyl cellulose (CMC; 0.5 ml) in 0.1 M sodium phosphate buffer (pH 7.0) at 37 °C for 30 min. DNS reagent (3 ml) was added to stop the reaction mixture which was boiled for 5 min in hot water bath. The absorbance was measured at 540 nm. One unit of enzyme activity was defined as the amount enzyme that liberates 1 μmol of glucose per millilitre per minute under specified condition [6].

### Scanning electron microscopy

Scanning electron microscopy (SEM) was performed to understand the ultrastructural changes of untreated and treated biomass. The images were processed using gold sputter to get qualitative details of structural changes.

### Fourier transform infrared spectroscopy

Fourier transform infrared spectroscopy (FTIR) was performed to understand the biochemical properties of untreated and pre-treated enzyme hydrolysed substrate. Samples were scanned within a range of 500–4000 cm^−1^.

## Results

### Enzyme source

Cellulase enzyme extracted from *Bacillus cereus* showed an activity of 62 IU/g of dry cellulase enzyme.

### Pre-treatment of plant material

**Fig. 1:**
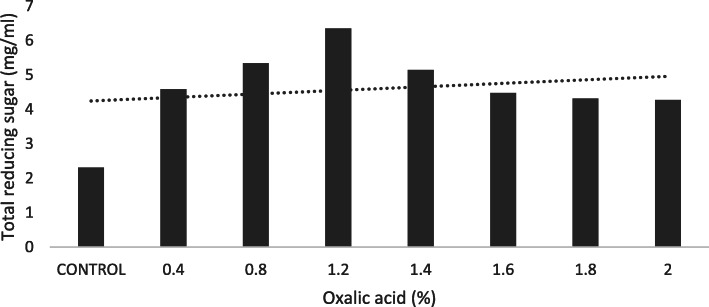
Total reducing sugar (mg/ml) yield for respective OAA (%) at standard SC (1%). Dotted line represents the projected trend

**Fig. 2:**
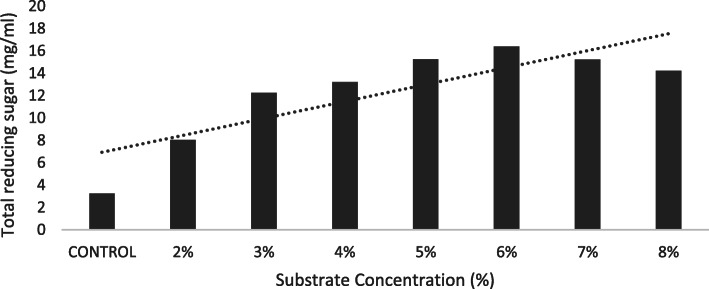
Total reducing sugar (mg/ml) yield for SC (%) at standard OAA (1.2%). Dotted line represents the projected trend

**Fig. 3:**
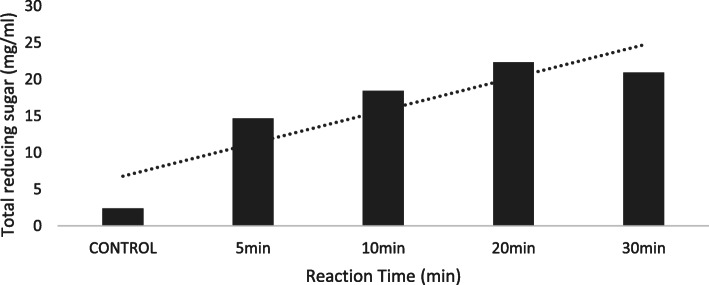
Total reducing sugar (mg/ml) yield for respective reaction time (min). Dotted line represents the projected trend

The highest reducing sugar yield (6.35 mg/ml) was observed at 1.2% of oxalic acid concentration (Fig. 1). Substrate concentration of 6% produced 16.32 mg/ml total reducing sugar (Fig. 2). Reaction time of 20 min (120 °C) recorded 22.32 mg/ml reducing sugar using oxalic acid pre-treatment under optimized condition (Fig. 3).

### Optimization of enzymatic saccharification

**Fig. 4:**
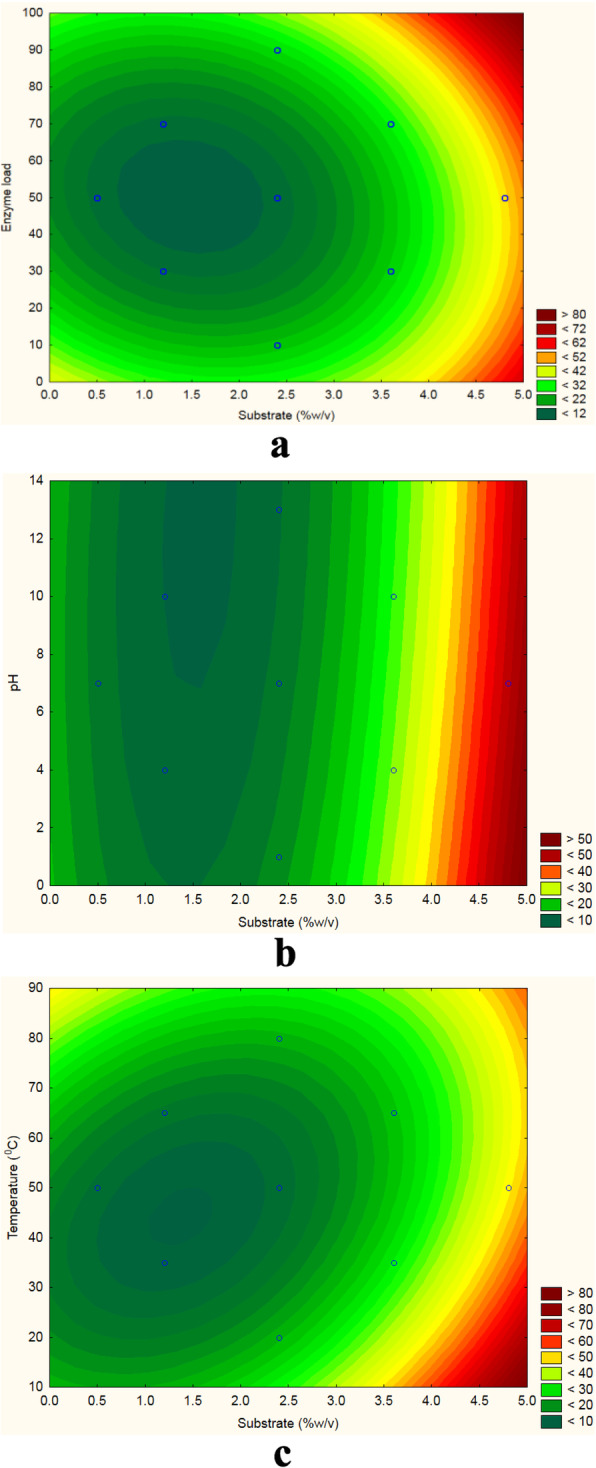
Two-dimensional contour response surface plots showing the mean effects independent variables and their interaction on reducing sugars (mg/g) for **a** enzyme load vs substrate, **b** pH vs substrate and **c** temperature vs substrate

**Fig. 5:**
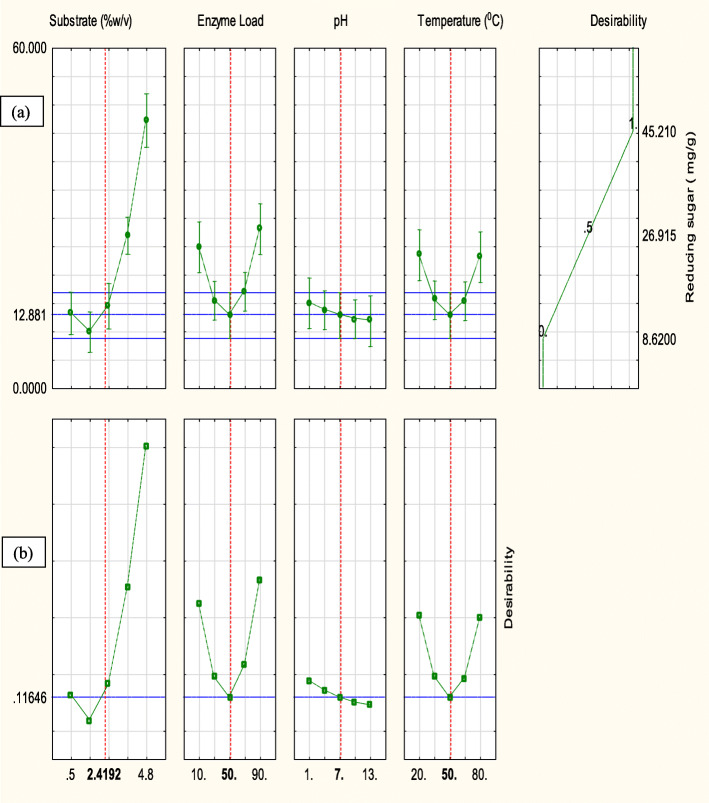
**a** Desirability profile. **b** Model builder prediction for yield of reducing sugars using response surface methodology

Enzymatic saccharification showed significant results (*p* < 0.0001; *R*^2^ = 0.96) (Table 2). RSM revealed that the yield gradually increased with rise in temperature (20–50 °C), SC (0.5–4.8% w/v) and enzyme load (10–50 IU/g) (Fig. 4a–c). Contrary to the experimental value of 42.13 mg/g (SC—2.4%; enzyme dosage—50 IU/g; pH 7.0; 50 °C), desirability and prediction model forecasted 45.21 mg/g of yield (Fig. 5a, b).

**Table 2:** Analysis of variance for the model regression for yield of reducing sugars

Factors	Sum of squares	Degree of freedom	Mean square	*F* value	Significance
Substrate (%w/v) (L)	1200.478	01	1200.478	172.2361	0.000000****
Substrate (%w/v) (Q)	318.281	01	318.281	45.6648	0.000031****
Enzyme load (L)	14.586	01	14.586	2.0927	0.175892
Enzyme load (Q)	212.384	01	212.384	30.4713	0.000181****
pH (L)	14.774	01	14.774	2.1196	0.173363
pH (Q)	0.381	01	0.381	0.0546	0.819471
Temperature (°C) (L)	0.051	01	0.051	0.0074	0.933150
Temperature (°C) (Q)	125.065	01	125.065	17.9435	0.001398***
1L by 2L	21.739	01	21.739	3.1189	0.105088
1L by 3L	2.024	01	2.024	0.2903	0.600751
1L by 4L	91.250	01	91.250	13.0919	0.004039 ***
2L by 3L	66.872	01	66.872	9.5943	0.010150**
2L by 4L	54.723	01	54.723	7.8513	0.017213**
3L by 4L	12.763	01	12.763	1.8311	0.203152
Error	76.670	11	6.970		
Total SS	2352.011	25			

*R*^2^ = 0.96

*Significant differences (*p < 0.05, **p < 0.01, ***p < 0.001, ****p < 0.0001)

### SEM

**Fig. 6:**
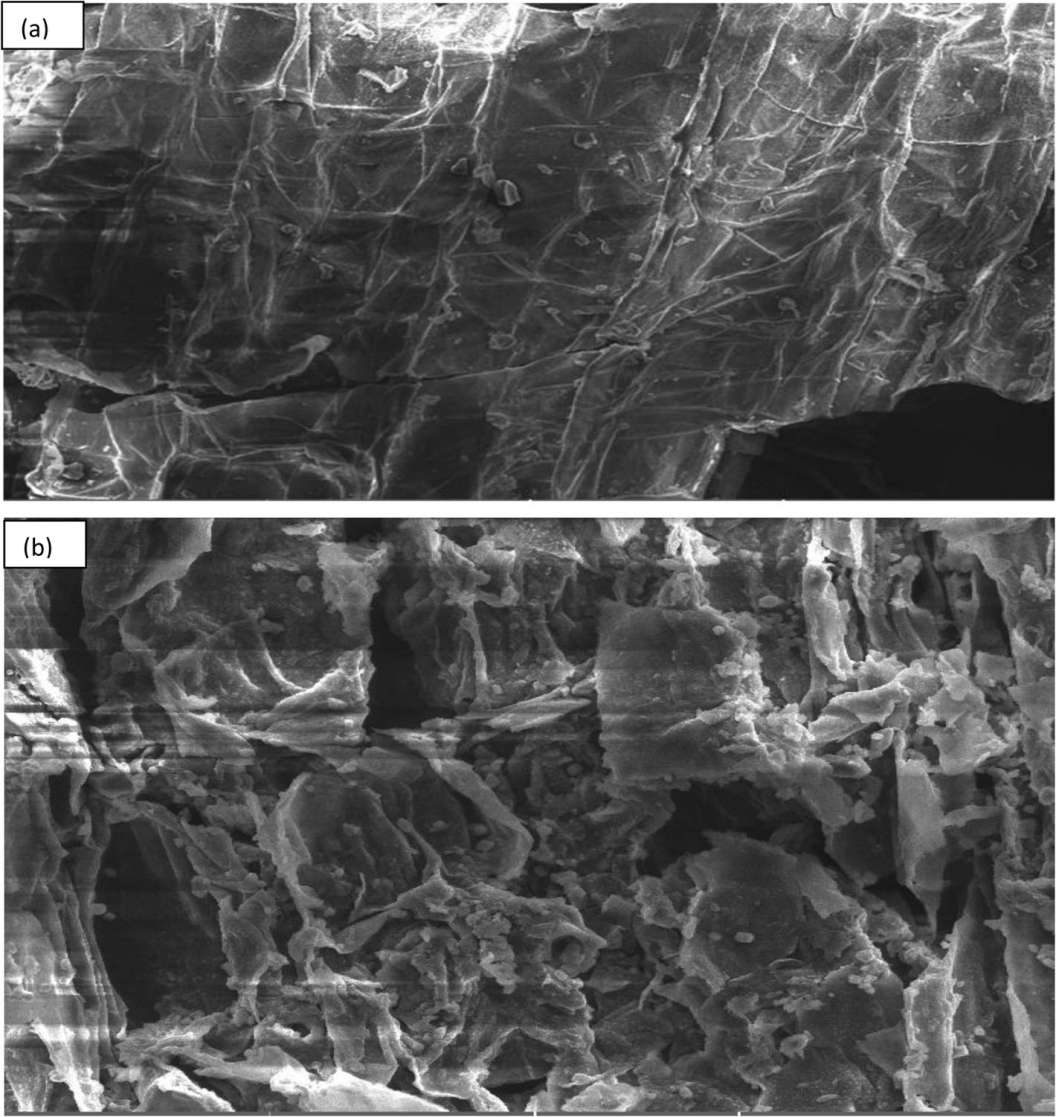
Scanning electron micrograph. **a** Untreated. **b** Pre-treated and enzyme hydrolysed sample

SEM revealed the untreated sample as a flat, regular and compact structure. The hydrolysed sample exhibited disorganized patterns, corroded and internal fibres of cell wall components. Display of porosity on the surface of the treated sample indicated successful hydrolysis [2, 33] (Fig. 6a, b).

### FTIR

**Fig. 7:**
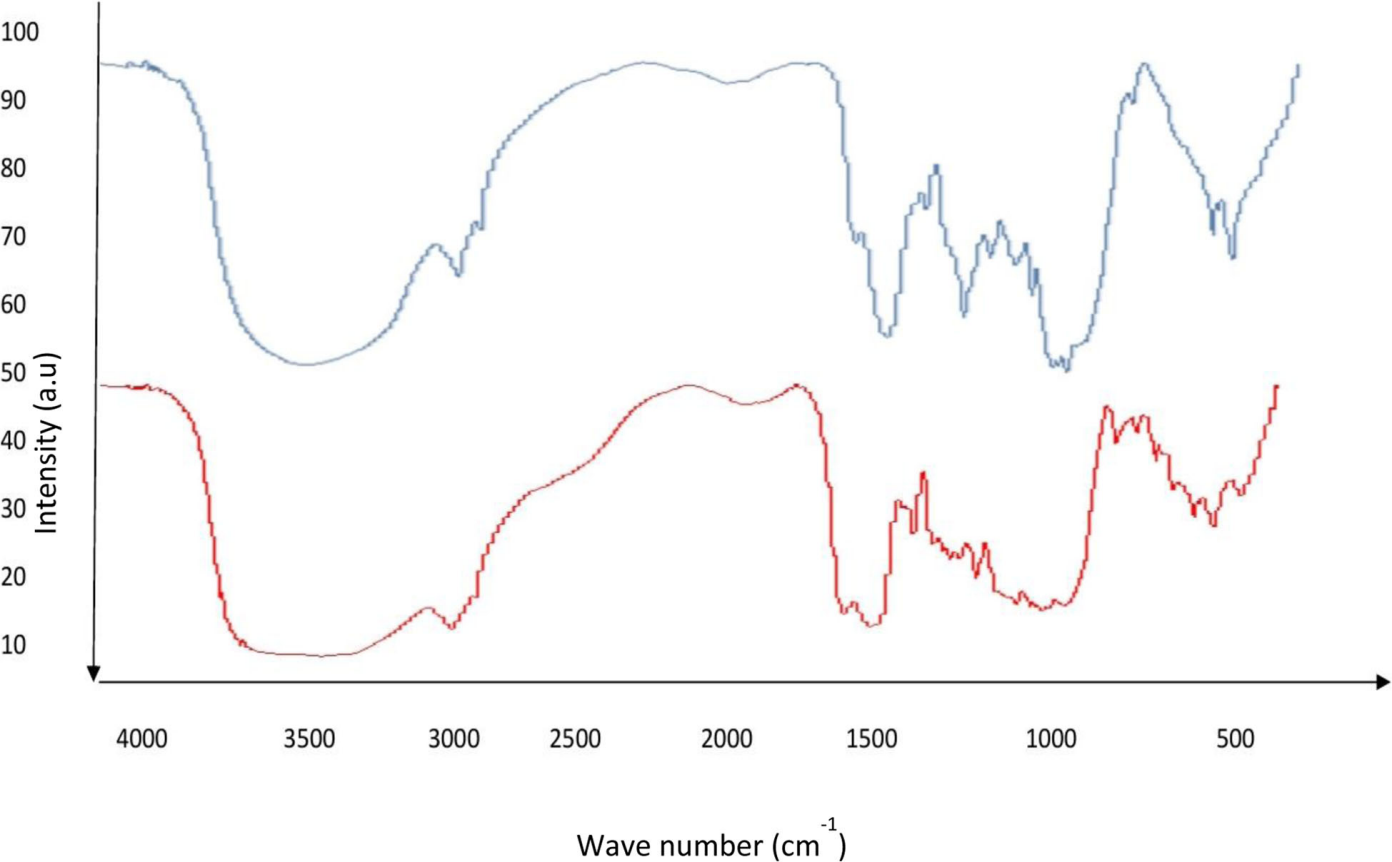
FTIR. Red colour—untreated biomass; blue colour—pre-treated and enzyme hydrolysed biomass

The band stretching at 890 cm^−1^ corresponded to the glycosidic deformation C–H bond and 1750 cm^−1^ to asymmetric and symmetric bond in lignin and cellulose [4]. Xu et al. [29] reported the wavelength range of lignin in rice straw from 1000 to 2500 cm^−1^ and yellow poplar from 800 to 2500 cm^−1^. Similarly, we observed the wavelength between 3000 and 4000 cm^−1^ which corresponds to lignin (Fig. 7). It is evident that the lignin composition varies in plant biomass.

## Discussion

Aquatic weeds unpleasantly occupy a major portion of the aquatic ecosystem. Therefore, research regarding generation of biofuels from them has been quite popular. This is evident because of a huge quantity of cellulose content in them. The utilization of their biomass ultimately depends on first, the extraction of sugars, and secondly, the exploitation of the fermentable capacity of extracted sugars for maximal ethanol generation [14, 32]. The present work is aimed at achieving the maximal sugar production under optimal and economic conditions.

### Pre-treatment of plant biomass

Rattanaporn et al. [21] have used OAA pre-treatment to get an elevated sugar yield of 1.7 mg/ml in liquid phase thereby enhancing enzymatic saccharification. Vanegas et al. [27] used 6% of OAA pre-treatment on *Laminaria digitata* and *Saccharina latissimi* which released approximately 21 and 24 mg/ml of reducing sugar, respectively. Lee and Jeffries [13] suggested that oxalic acid belongs to dicarboxylic acid which yields high monomer sugar and produces a smaller number of inhibitors in hydrolysate. Indeed, the mentioned studies throw light on the applicability of the OAA pre-treatment and substantial yield of sugar. Correspondingly, we found the sugar yield of 6.35 mg/ml after using 1.2% oxalic acid.

We noted an increased yield up to 22.32 mg/ml at 120 °C and reaction time of 20 min. Since maximum reaction time is invested behind substrate conversion, sugar concentration is proportional to degradation time [20]. Higher biomass causes mechanical delay, uneven mix up of substrate and availability of water molecule to disintegrate lignin, hemicellulose and cellulose [17]. Lower temperatures and short reaction time (using concentrated sulphuric acid) have known to degrade sugars and release levulinic acid which inhibits ethanol production during fermentation [8, 12, 26].

### Optimization of enzymatic saccharification

Maximum sugar yield is obtained at optimum conditions, whereas lower yield is due to unfavourable condition which has been previously reported by Yoonan and Kongkiattikajorn [30]. In combination, enzyme dosage ratio and temperature have known to influence sugar yield [16]. Our study revealed temperature and enzyme-substrate ratio to be the most important factors influencing the sugar yield during enzymatic saccharification.

Krishna and Chowdary [9] observed a maximal saccharification rate at 50 °C, which supports our study in that the maximum yield was noted at enzyme load 50 IU/g and temperature 50 °C, liable for sugar production. Beyond this condition, decline of hydrolysis rate, thermal deactivation, loss of enzyme adsorption and enzyme inhibition were reported by Robinson [23].

In our study, substrate concentration and enzyme dosage of 2.4% and 50 IU/g, respectively, showed a better fit for the releasing sugar. As substrate concentration and enzyme activity are inversely related, eventually, a saturation point will descend, where all the available enzyme will get exhausted thereby increasing the substrate concentration. Therefore, there will be no effect in hydrolysis, and hence, reduction in saccharification and sugar production is expected to occur in this situation [1].

In our study, pre-treated material underwent better hydrolysis of the native biomass structure. This enhanced the efficiency of enzymatic saccharification yielding high amount of sugar, which is similar to hydrolysis of sugarcane bagasse [15]. We observed maximum saccharification at substrate concentration of 2.4%, which depends on enzyme-substrate synergism. Higher substrate concentration inhibits the saccharification process [33].

Gregg and Saddler [7] suggested that 2% substrate concentration, 49.56 IU/g cellulase dosage and 50 °C are the best conditions for maximum yield. Their results corroborate our study since the influencing parameters for sugar production were in approximation with their outcomes.

## Conclusion

The present work aims to enhance reducing sugar production from *Typha latifolia* using pre-treatment and enzymatic saccharification. In our study, the total reducing sugar content amounted to 22.32 mg/ml under pre-treated optimum conditions (OAA—1.2%; SC—6%; RT—20 min), while enzymatic saccharification yielded 45.21 mg/ml of reducing sugar (SC of 2.4%, enzymatic dosage—50 IU/g; pH 7.0; temp—50 °C). Rapid industrialization has seen a rise in energy demands, and preference has always been given to cost-effective strategies of energy generation. We attempted an effective economical method for bioconversion of this aquatic weed biomass into sugar content, thereby making it a potential substrate for bioethanol production.

## Data Availability

The authors declare that all generated and analysed data are included in the article.

## Abbreviations

OAA: Oxalic acid
SC: Substrate concentration
RSM: Response surface methodology
OVAT: One variable at a time
CCD: Central composite design
SEM: Scanning electron microscopy
FTIR: Fourier transform infrared radiation
IR: Infrared radiation
